# Phasic Oscillations of Extracellular Potassium (K_o_) in Pregnant Rat Myometrium

**DOI:** 10.1371/journal.pone.0065110

**Published:** 2013-05-28

**Authors:** Roger C. Young, Gabriela Goloman

**Affiliations:** Department of Obstetrics, Gynecology and Reproductive Sciences, University of Vermont, Burlington, Vermont, United States of America; University of Chicago, United States of America

## Abstract

K-sensitive microelectrodes were used to measure K^+^ within the extracellular space (K_o_) of pregnant rat myometrium. Contractile activity was monitored by measuring either force or bioelectrical signals. Single and double-barreled electrodes were used. Double-barreled electrodes allowed monitoring of electrical activity 15 microns from the site of K_o_ measurement. From double-barreled electrode experiments, the bioelectrical burst started first, and then K_o_ began to rise 0.6 ± 0.1 seconds later. This delay indicates that K^+^ leaves the cells in response to local electrical activity rather than vice versa. Four control experiments were performed to assess the influence of electrical artifacts caused by tissue motion on K_o_ values. When observed, artifacts were negative and transient, and hence would result in an underestimation of K_o_ rises. Artifacts were minimized when tissue motion was minimized by fixing the tissue at both ends. At 37°C, 7 single barreled experiments and 45 contractions were analyzed. Resting K_o_ was within 1 mM of bath K^+^ (5 mM) at the beginning and end of the experiments. K_o_ rose during the contraction, fell after the completion of the contraction, and normalized before the next contraction began. Peak K_o_ values observed during force production were 18.8 ± 5.9 mM, a value high enough to modulate tissue-level electrical activity. K_o_ required 15.7 ± 2.8 seconds to normalize halfway (t_50_). Six experiments expressing 38 contractions were performed at 24°C. The contraction period was longer at 24°C. Values for peak K_o_ (26.2 ± 9.9 mM) and t_50_ (29.8±16.2 sec) were both larger than at 37°C (p<0.0003 for both). The direct relationships between peak K_o_, t_50_ and the contraction period, suggest elevations in K_o_ may modulate contraction frequency. The myometrial interstitial space appears to be functionally important, and K_o_ metabolism may participate in cell-cell interactions.

## Introduction

In the 1960s Anderson[1] discovered that tissue-level electrical activity is expressed in myometrial tissue strips. It is now generally accepted that tissue-level contractions are caused by the expression of tissue-level electrical activity and excitation-contraction coupling[2], [3]. Over the ensuing decades, investigators elucidated many of the mechanisms of myometrial electrical excitability. A large body of work is now available that details the complex interactions of cell-based systems that are necessary to generate contractions, including a mathematical model of excitation-contraction coupling for myocytes[4]. However, despite a deep understanding of cellular excitability, gaps remain in our understanding of how the electrical mechanisms of the myocyte relate to excitability of the tissue [5]. One interesting hypothesis is that electrical excitability at the tissue-level may in part be regulated by metabolic processes[6]. An example of one such mechanism would be if phasic myometrial contractions caused changes of the ionic composition of the extracellular space.

Potassium in the extracellular space (K_o_) can be quantitatively measured using K-sensitive microelectrodes, and changes in K_o_ have been reported in other tissues. Small rises in K_o_ are seen in the brain with photostimulation of the retina[7], but very large rises can be found under pathological conditions[8]. The mechanism of the vascular myogenic response[9] involves elevation of K_o_ [10], [11], although other mechanisms likely contribute[12]. Exercise causes a large release of K^+^ from skeletal muscle[13]. In cardiac tissue K_o_ rises by 0.5 to 1.5 mM with each contraction[14] and by 3–4 mM in Purkinje tissue[15], although K_o_ accumulates to much larger values when these tissues are artificially paced faster than K_o_ can normalize. In this work, we will for the first time use K-sensitive electrodes to observe phasic rises in K_o_ in contracting pregnant myometrium.

## Materials and Methods

### Pregnant rat myometrium

This study was carried out in strict accordance with the recommendations in the Guide for the Care and Use of Laboratory Animals of the National Institutes of Health. The protocol was approved by the Committee on the Ethics of Animal Experiments of the University of Vermont (IACUC protocol #07–055AP). All efforts were made to minimize suffering. Timed pregnant rats were purchased from Charles River and used between d 20 and 21 gestations. Rats were euthanized using pentobarbital and decapitation, and myometrial tissue harvested. Full thickness myometrial strips, ∼1.5 mm wide and 1 cm long, were cut parallel with the longitudinal muscle.

### Horizontal experimental chamber, experimental conditions

Full thickness myometrial strips were mounted horizontally in an experimental chamber and stretched ∼30%. Where indicated, force measurements were performed by securing one end of the tissue to the chamber and the other to a Grass FT-03 force transducer.

Some experiments were performed with bath flow. Bath solutions for these experiments were (in mM) 120 NaCl, 5 KCl, 1.8 CaCl_2_, 0.5 MgCl_2_, 25 NaHCO_3_, 11 glucose, pH 7.4, saturated with 95% O_2_, 5% CO_2_. Flow rates were 1–2 ml/min through a chamber with a 3.5 ml volume, providing a bath exchange time of 2 to 4 minutes.

Some experiments were performed under conditions designed to minimize tissue movement and reduce motion artifacts. Specifically, the bath solution was not flowed through the chamber (2 ml volume) when the tissue was secured at both ends. Eliminating the force transducer eliminated the movements of the spring arm, but made it impossible to directly measure force. To determine the timing of contractions without measuring force, we measured bioelectrical activity with contact electrodes (see Measuring AC bioelectrical signals, below). Under no-flow conditions bath solutions were (in mM) 135 NaCl, 5 KCl, 1.8 CaCl_2_, 0.5 MgCl_2_, 10 Na-HEPES, 11 glucose, pH 7.4. Experiments were performed at 37±1°C and 24 ± 1°, and bath temperatures were monitored using a thermocouple. For experiments at 37°C, temperature was elevated by heating the chamber and preheating the bath solutions prior to exchange. Room temperature experiments were performed without heating.

### Measuring DC voltages

Voltages were recorded using one or two A-M systems model 3000 high impedance amplifiers set to the DC mode. A silver chloride-coated wire was placed in the bath and used a reference. The bath ground was also connected to system ground of the head stages of the amplifiers. Signals were filtered at 1 kHz, digitized, stored on a personal computer, and analyzed using AD Instruments Chart 5 software.

### Construction of K-sensitive electrodes

K-sensitive electrodes were constructed using methods similar to those of Obrocea and Morris[16]. Single-barreled pipettes were pulled from filamented capillary tubes in a manner similar to pulling patch clamp pipettes. Tip openings of single-barreled electrodes were 1–2 µm and, prior to being made K-sensitive, had impedances of 5–10 MΩ when filled with 200 mM KCl.

The first step in construction of K-sensitive electrodes was silanization of the pipette tip. Initially, pipettes were filled to the tip with 200 mM KCl. Tips were then dipped in chlorodimethylsilane (Sigma-Aldrich) solution and positive pressure was applied with a syringe, which pushed a drop of the aqueous KCl solution into the organic solvent. Suction was then applied for 3–5 seconds, which brought chlorodimethylsilane into the tip. This process was repeated 3 times, ending with application of positive pressure to expel the organic solvent. Finally, potassium-ionophore 1- cocktail A solution (a valinomycin-based K^+^ sensitive exchanger, Sigma-Aldrich) was introduced into 10 to 100 µm into the tip by application of suction for 3–5 seconds. Impedances of the K-sensitive electrodes were typically 22 to 28 Mohm. Prior to use, all electrodes were stabilized in 5 mM KCl solution for at least 30 minutes, zeroed in 2.5, then tested in 5, 10, 20, and 40 mM KCl solution. Voltage drifts in test solutions were less than 0.5 mV/minute. For each experiment, the response of each electrode to test solutions was used to convert observed voltage changes to changes in K_o_.

Some single-barreled electrodes were constructed to serve as negative control electrodes, and were prepared by omitting introduction of potassium-ionophore solution. These “non-K-sensitive” electrodes were designed to be unresponsive to changing KCl, which was confirmed by testing in the 2.5 to 20 mM KCl test solutions.

K-sensitive electrode measurements were performed at 24° and 37°C. We anticipated a small (4%) difference of electrode response at these temperatures. However, because we were unable to detect measurable differences in individual electrode responses to test solutions at these two temperatures, we did not correct for temperature changes during data analysis.

### Fabrication of double-barreled electrode: one K^+^-sensitive, one K^+^-insensitive

Double-barreled electrodes were made from filamented double capillary tubes (Sutter Instruments, 2BF-150-86-10). After pulling, one capillary tube was then shortened by 1–2 cm by first carefully notching one tube with a glass cutter, then breaking the glass connection between the capillary tubes. The end result was a double-barreled pipette with one back length shorter than the other.

The short electrode was back-filled with bath solution and wrapped with parafilm. The longer electrode was back-filled with 200 mM KCl, and placed in a patch-clamp electrode holder. The pressure/suction procedure described above was then used to silanize and tip-fill the long electrode with potassium-ionophore 1- cocktail A solution. The tips were maintained in a beaker containing bath solution until used.

### Measuring AC bioelectrical signals

Bioelectrical signals were obtained using contact electrodes as previously reported[17]. Contact electrodes were made from 0.7 mm ID glass capillaries containing a silver chloride-coated silver wire. The electrodes filled with bath solution by capillary action when placed into the chamber and onto the tissue. Tissue-level bioelectrical signals were monitored by using one of the A-M systems model 3000 high impedance amplifiers in the AC mode. Raw data were recorded using high pass filters set at 1 Hz, low pass set at 1 kHz, and a 60 Hz notch filter. The contact electrodes recorded spike-like bioelectrical signals which were associated with the force-producing phase of each contraction. Even though the bioelectrical spikes occurred at frequencies of 1–2 Hz, the spike widths were ∼40 msec (25 Hz). To reduce noise and spurious peaks, raw data were routinely digitally filtered (low pass 30 Hz) for data analysis. Using this frequency window there was minimal loss of fidelity of the bioelectrical signal and the experimental time resolution was ∼200 msec. When measuring time intervals between the start of bioelectrical activity and rises in K_o_, raw data were low pass filtered at no less than 50 Hz to improve time resolution to better than 100 msec.

### Using K-sensitive electrodes while simultaneously measuring bioelectrical signals with a separate contact electrode

As described above, rat myometrial tissue strips were mounted in the chamber and bioelectrical signals recorded with a contact electrode. Using a separate micromanipulator, a K-sensitive electrode was zeroed in the bath (KCl = 5 mM), then placed into the tissue at a distance of 2–3 mm from the contact electrode.

After initial placement, the K-sensitive electrode typically drifted ∼3–4 mV every 10 minutes. The amount of drift was confirmed at the conclusion of each experiment by removing the electrode from the tissue and checking the potential of the bath relative to a standard K^+^ solution. Rather than periodically removing the electrode from the tissue and re-zeroing, corrections for drift were made by measuring the voltage changes using the voltage immediately prior to each contraction as reference. In this manner it was possible to obtain recordings of long duration and many contractions without re-zeroing and repositioning the electrode.

### Using double-barreled K-sensitive electrodes while simultaneously measuring force

Double barreled electrodes were placed in an electrode holder, and the amplifiers of both electrodes were initially placed in the DC mode. After zeroing the potentials in the bath solution, the electrode was placed into the tissue. In some experiments, local bioelectrical activity was assessed by switching the amplifier of the non-K-sensitive electrode to the AC mode.

### Statistics

Average values and standard deviations are reported. Comparisons between data sets were made using the 2-tailed t-test for unpaired or paired data, as appropriate.

## Results

### Voltage responses of the K-sensitive electrode

**Figure 1:**
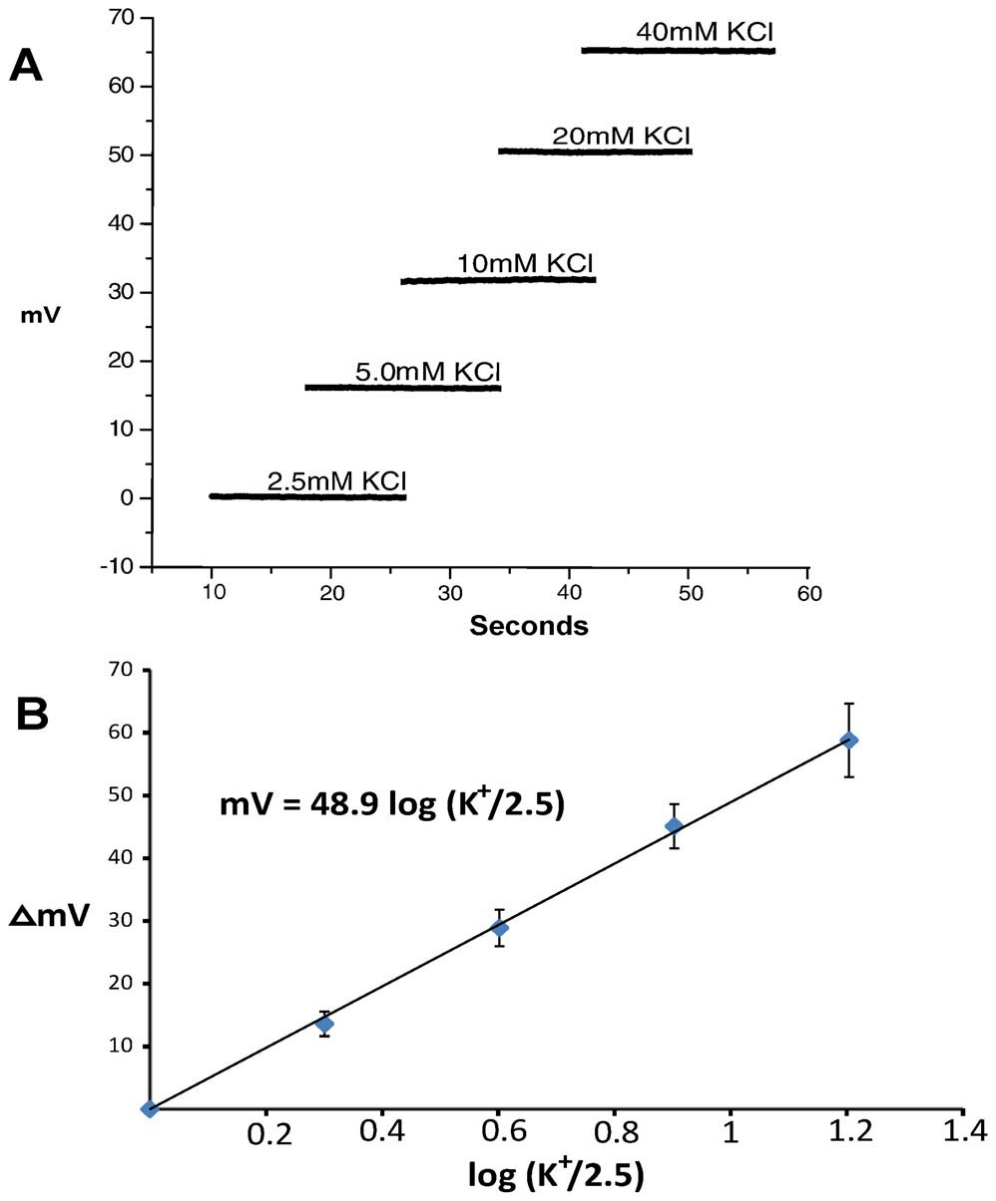
Determining the responses of K-sensitive electrodes. A) Voltage responses of a K-sensitive electrode in test solutions containing differing concentrations of KCl at 24°C. B) Logarithmic plots of voltage response of K-sensitive electrodes with changing KCl. The slope for these K-sensitive electrodes was 48.9 mV/decade; Error bars represent standard deviations.

After zeroing the K-sensitive electrode in 2.5 mM KCl solution, the electrode was placed in test solutions containing 5, 10, 20 and 40 mM KCl at 24°C. Using the negative terminal of the amplifier probe as bath ground, positive voltage changes were observed with increasing KCl (n = 5; Fig. 1A). Voltage responses were plotted against the logarithm of the ratio of the test solution K^+^ and 2.5 (Fig. 1B). The slope (48.9 mV/decade) is slightly lower than that predicted by the Nernst equation (58.9 mV), yet the linearity indicates the electrodes can quantify K^+^ from 5 to 40 mM. In practice, each electrode was tested prior to use in standard KCl solutions from 2.5 to 20 mM to determine the K^+^ sensitivity. We were unable to find any difference in the responses of individual electrodes between 37° and 24°C (see Figure S1), so we did not correct electrode responses for changes of temperature. Non-K-sensitive electrodes, constructed by omitting the potassium-ionophore solution, were insensitive to K^+^.

### Single K-sensitive electrode voltages and bioelectrical signals

In this series of experiments we sought to determine if changes in K_o_ were associated with myometrial contractions. To minimize tissue movement, the tissues were fixed at both ends and experiments were performed under no-flow conditions. Because forces were not measured, bioelectrical activity was used as a reporter for the timing of contractile activity. Bioelectrical activity can be observed with contact electrodes, and is a direct measure of the expression of tissue-level action potentials. We have previously demonstrated that rat myometrium expresses burst-like bioelectrical activity that is closely associated with force generation[17]. Thus, while this technique cannot quantify force production, we were able to precisely determine the beginning and end of each contraction with the first and last spikes of the bioelectrical bursts.

A K-sensitive electrode was placed into the bath, the voltage was zeroed, and then the electrode was placed into the tissue. After several minutes, recorded voltages were 0±5 mV in all cases, indicating that the K^+^ values at the location of the electrode tip were within ∼1 mM of bath K^+^ (5 mM).

**Figure 2:**
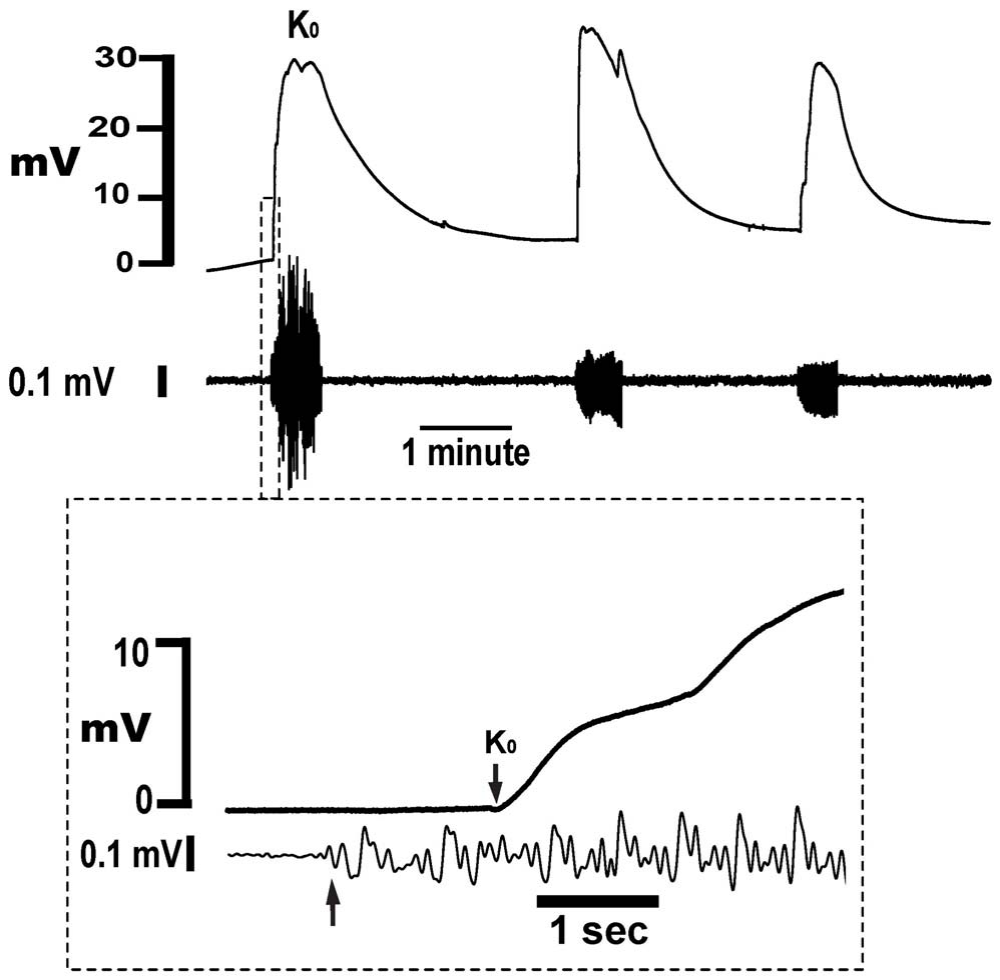
Simultaneous recording of voltage changes from a K-sensitive electrode and a loose contact electrode in pregnant rat myometrium. T = 37°C, bath KCl is 5 mM, no-flow conditions. Both ends of the tissue are securely held. Upper tracing: Voltage responses of K-sensitive electrode. Lower tracing: Bioelectrical activity, which represents the force-producing phase of the contraction. Insert – expansion of data at the beginning of the first contraction. Arrows indicate the onset of bioelectrical activity and beginning of the K_o_ rise, and the time between the arrows is the delay of the onset of K_o_.

With continued monitoring, transient voltage rises of the K-sensitive electrode were observed that were closely associated with burst-like bioelectrical activity (Fig. 2). K_o_ began to rise soon after the start of the bioelectrical activity (Fig. 2 insert), exhibited a pseudo-stable plateau during the expression of bioelectrical activity, then fell relatively slowly after the end of the burst. Although K_o_ returned to near baseline values before the onset of each contraction, K_o_ elevations extended into the period between contractions. Partly because the voltage changes we observed were so large, and partly because there are no prior reports of K_o_ elevations in myometrium, we performed a series of experiments specifically designed to address the possibility that the observed voltage changes were the result of electrical artifacts rather than rises in K_o_.

### Assessing electrical artifacts due to motion of the tissue

**Figure 3:**
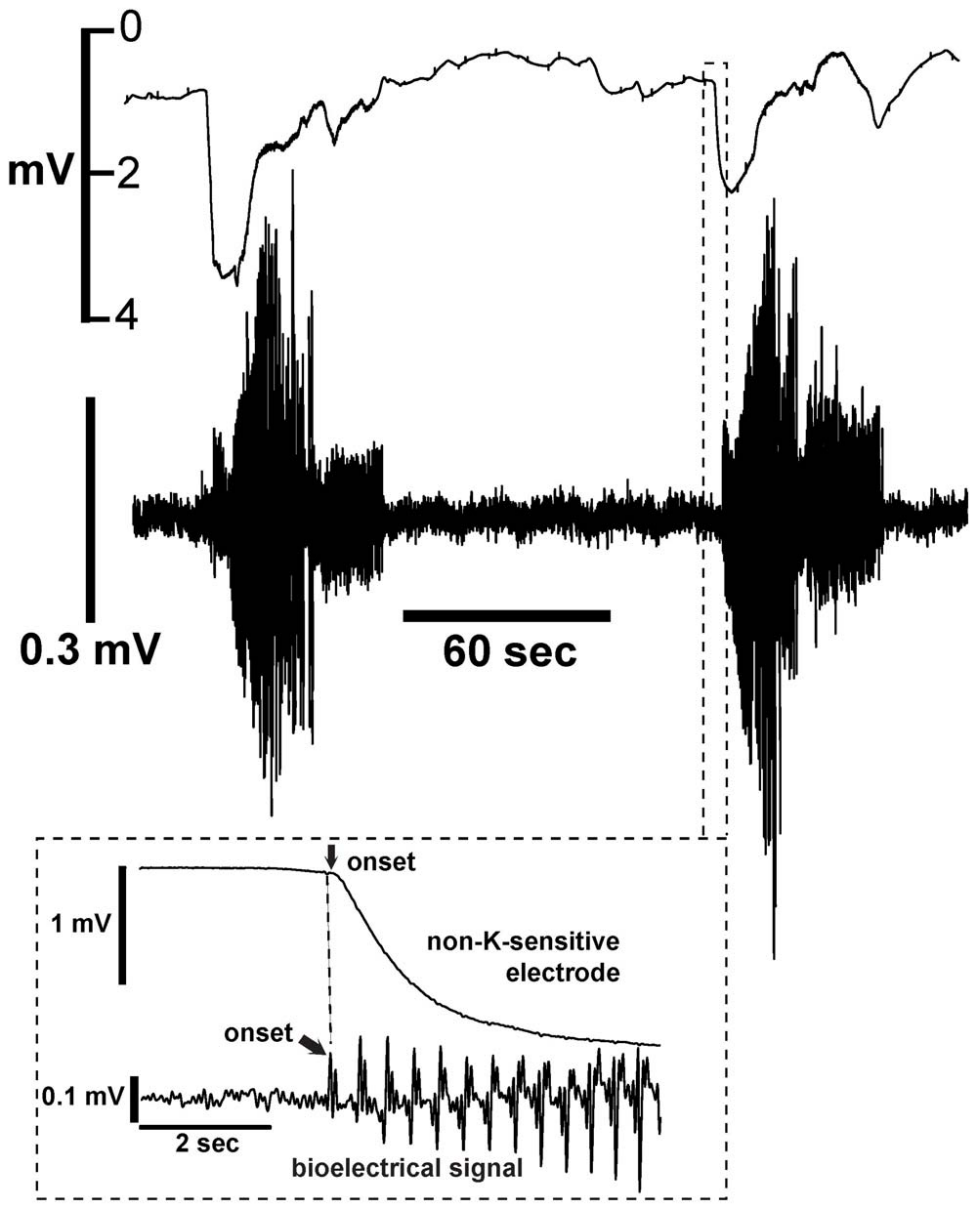
Upper tracing: Voltage responses of the non-K-sensitive electrode using the experimental conditions described in Figure 2. Lower tracing: Bioelectrical activity recorded using a separate contact electrode. Non-K-sensitive electrodes were constructed by omitting potassium-ionophore solution in the electrode. Insert - expansion of data at the beginning of the contraction. T = 37°C.

Even when held isometrically, myometrial strips move when they contract. As an initial test for artifacts, we recorded signals using non-K-sensitive electrodes (5 experiments; 18 contractions). Non-K-sensitive electrodes were prepared using the silanization procedure, but with omission of the K^+^-ionophore, cocktail A. As before, tissue strips were fixed at both ends, and bioelectrical signals were recorded with a contact electrode to determine the beginning and end of each contraction. In half the contractions, voltage changes associated with bioelectrical activity were not observed. In the other half, small voltage changes of variable magnitude could be identified (Fig. 3). When observed, the voltage changes were negative, transient, and less than 5 mV. These signals began near the onset of each contraction, and lasted less than 20 seconds. Because the voltage responses of the non-K-sensitive electrodes were always negative, we concluded that artifacts due to motion result in an underestimation of K_o_ rather than an overestimation.

### Testing for motion artifacts using double-barreled electrodes and a force transducer

**Figure 4:**
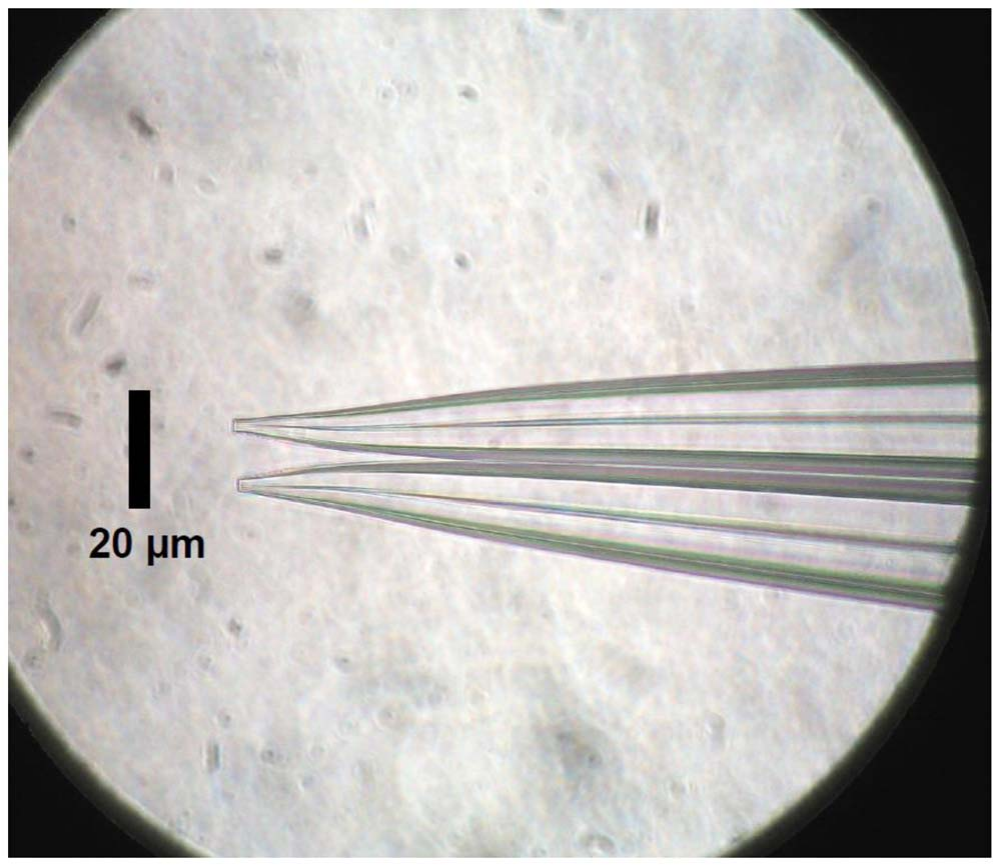
Tips of double-barreled electrode.

To further test for spurious voltages near the tip of the K-sensitive electrode, we used double-barreled electrodes. Using multiple heat-pull cycles on filamented double capillary tubes, we fabricated double-barreled electrodes with both tips similarly shaped to the single barreled electrode, but separated by 14 to 16 µm (Fig. 4). As detailed in Methods, the back-fill end of one of the capillary tubes was shortened and used as an open electrode, and the longer electrode was made K-sensitive. When filled with bath solution, the impedances of the short electrodes were 3.5 to 5.5 Mohm. The long barreled electrodes were silanized and made sensitive to K^+^ by addition of K^+^ ionophore solution to the tip. The impedances of these electrodes were 24 to 28 Mohm. Both amplifiers were initially set to the DC mode. Because electrical artifacts caused by tissue motion would be detected by the open electrode, we were able to measure force and still be confident that the increased tissue motion inherent to using a force transducer did not introduce unanticipated artifacts.

**Figure 5:**
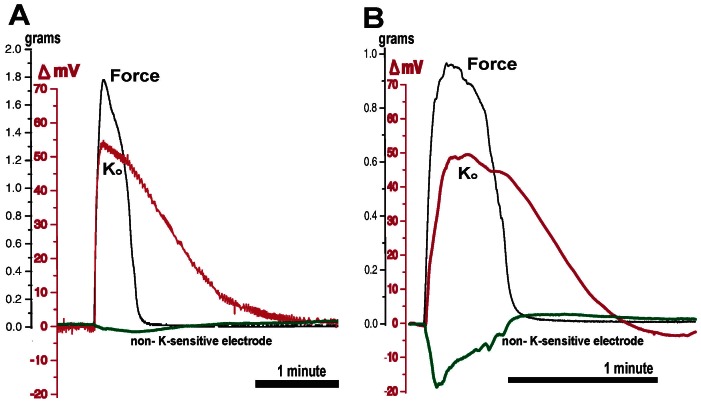
Double-barreled electrode + force tracing. The tip of the long electrode was made K-sensitive as described (red). The short barrel was untreated, filled with bath solution (green), and monitored in the DC mode. Experiments (A) and (B) are contractions from two different tissues that demonstrate the smallest (A, green) and largest (B, green) signals observed from the non-K-sensitive electrodes using double-barreled electrodes. K_o_ normalized on a much longer timescale than the decay of force, and persisted much longer than the voltage artifact demonstrated by the non-K- sensitive electrode. Data obtained under flow conditions, T = 37°C.

Three experiments were performed on three different tissues and 19 contractions were analyzed. With each contraction, the non-K-sensitive electrodes displayed negative voltage deflections of varying magnitudes (Fig. 5). The smallest deflection (A) was -3 mV, and the largest (B) transiently approached -20 mV. The defections closely mirrored force production, and rapidly returned to 0 mV near the end of the contraction. As with the single barreled non-K-sensitive electrodes, no positive voltage deflections were observed from the non-K-sensitive half of the double-barreled electrode.

The K-sensitive halves of the double-barreled electrodes demonstrated voltage changes similar to those observed using the K-sensitive single barreled electrodes. Rises in K_o_ were closely associated with force production, and K_o_ elevations persisted well into the period between contractions.

### Testing for artifacts using tissue stretch

To complete our testing for motion-induced electrical artifacts, we mechanically stretched the tissue using the force transducer and the double-barreled electrode. The purpose of this experiment was to determine if the K-sensitive electrode responds to large tissue displacements in the absence of a contraction. Since stretching myometrium can initiate contractions[18], it was necessary to use the force transducer to demonstrate the experimental stretch did not cause the tissue to contract. The non-K-sensitive electrode was used in the AC mode to monitor for local electrical activity.

**Figure 6:**
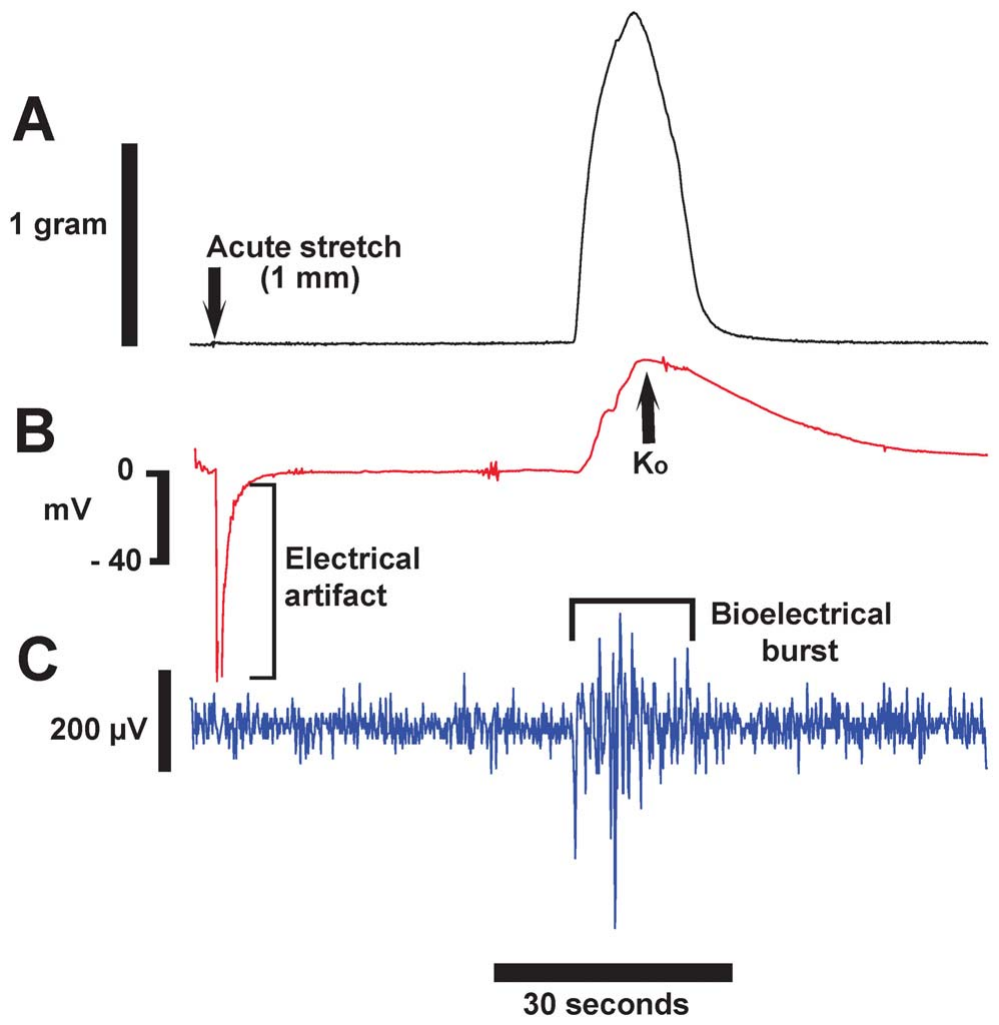
Mechanical stretch of tissue using the double-barreled electrode + force apparatus. Tissue was mechanically stretched 1 mm using a micrometer drive over 2–3 seconds. The stretch did not induce a contraction (A) and did not result in localized bioelectrical activity (C). A large negative deflection of the K-sensitive electrode (B) was observed for 3–4 seconds after beginning the stretch, but recovery to 0 mV was rapid and no positive voltages associated with stretch were observed. After 45 seconds, a spontaneous contraction occurred, and positive responses were observed from both the K-sensitive, and the bioelectrical sensing halves of the double-barreled electrode. Data obtained under flow conditions, T = 37°C.

After electrode placement, we verified each component was functioning by simultaneously observing force, K_o_ rises, and bioelectrical signals. Then, between contractions, we mechanically stretched the tissue by 1 mm over 2–3 seconds using a micrometer drive (Fig. 6, arrow). From the specifications of the FT-03 transducer, we approximated that during the peak of a contraction the spring of the transducer moved approximately 100 µm. We elected to stretch the tissue 1 mm in order to exceed what the tissue would normally experience and ensure the tissue moved by at least 100 µm along most of its length.

In Figure 6, stretch was not associated with an increase of force or bioelectrical activity, indicating a contraction was not initiated and no local electrical activity was induced (Fig. 6A, C). However, a very large negative voltage deflection of the K-sensitive electrode was associated with tissue movement (Fig. 6B). The deflection recovered to baseline within 3–5 seconds. Importantly, no large positive voltages were observed in the seconds after the completion of the stretch-induced electrical artifact. Within a minute after the stretch, the tissue spontaneously contracted, as revealed by the force tracing. Contraction-associated rises in K_o_ and bioelectrical activity were observed, confirming correct functioning of the double-barreled electrode. As before, these data indicate that in the absence of a contraction or regional electrical activity, movement of the tissue causes a negative voltage deflection of the K-sensitive electrode. These data confirm that artifacts due to tissue motion do not increase our reported values of K_o_.

### Cause-effect relationship between bioelectrical activity and the onset K_o_ rises

The purpose of this series of experiments is to determine if rises in K_o_ initiates electrical activity, or if electrical activity causes K_o_ rises. In Figure 2 we observed that bioelectrical activity started before the onset of K_o_ rises. To optimize the temporal resolution of the experiment, we minimized tissue motion by performing these experiments with both ends of the tissue fixed and without bath flow. We also measured bioelectrical activity with a separate contact electrode, which tends to further reduce tissue motion. We analyzed 45 contractions from 7 experiments and found bioelectrical activity always preceded the onset of K_o_ rises, with an average interval of 0.9 ± 0.5 seconds.

However, those data were obtained using surface electrodes placed 2–3 mm distant from the K-sensitive electrode. Because of this separation, the bioelectrical signals may not have precisely reflected the start of the electrical activity at the tip of the K-sensitive electrode. We therefore analyzed the data obtained using the technique described for Figure 6 (force plus double-barreled electrodes with the non-K-sensitive electrode monitored in the AC mode, and under bath flow conditions). Since the tips of the double-barreled electrode are only ∼15 microns apart, the observed bioelectrical activity closely reflects the expression of action potentials at the site K_o_ was monitored.

In three tissues, and 16 contractions, the first spike of the bioelectrical activity always occurred before the beginning of the K_o_ rises. The average time delay was 0.6±0.1 seconds. These data confirm that the local action potential occurs before K_o_ begins to rise. This directly indicates that rises in K_o_ are caused by expression of electrical activity rather than vice versa. Additionally, the relatively short delay between the start of the bioelectrical activity and the start of the K_o_ rises indicates that K^+^ begins to enter the extracellular space very soon after expression of the local the action potential. Since myocytes are the only reasonable source of K^+^, this implies that outward K^+^ currents are not restricted to the repolarization phase of the action potential.

### Quantifying K_o_

To convert voltages of the K-sensitive electrode to K_o_ values, we used the equation: 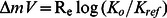
(1)


R_e_ is the response of the electrode to test solutions, and ΔmV is the observed voltage change above baseline (defined as the voltage immediately prior to the contraction). Since our initial data indicated resting K_o_ is 5±1 mM (e.g. the bath K^+^ concentration), we assigned the reference (K_ref_) value to 5 mM. This method of converting of ΔmV to K_o_ compensates for electrode drift that occurs with prolonged recordings, provided K_ref_ remained constant.

To determine if there were long-term changes in K_ref_ over time, we removed the K-sensitive electrode from the tissue after the decay of K_o_ was completed, but before the start of another contraction. This maneuver directly compared K_ref_ in the tissue with the 5 mM K^+^ of the bath. In 5 experiments where the tissue had been studied for at least 60 minutes, the voltage changes observed were near the resolution of the amplifier, and none was more than 3 mV. This indicates that large changes in K_ref_ did not occur, even in long experiments, and equation 1 could be used to quantify K_o_.

### Peak values of K_o_ at 37° and 24°C

In this series, we sought to quantify the magnitude of the K_o_ rises that are associated with contractile activity at two temperatures. To minimize artifacts of tissue movement, we secured both ends of the tissue to the chamber, and did not use bath flow. We used single barreled K-sensitive electrodes, and monitored bioelectrical activity with separate contact electrodes to determine the onset and offset of each tissue-level contraction. K_o_ values were calculated using Equation 1, and peak K_o_ is defined as the maximum value of K_o_ observed during each burst of bioelectrical activity. We performed 7 experiments and analyzed 45 contractions at 37°C (Table 1).

**Table 1:** K_o_ and contraction duration parameters at 37° and 24°C.

T (°C)	Peak K_o_ (mM)	t_50_ (seconds)	Burst duration (seconds)	Time between contractions (seconds)	
37	18.8 ± 5.9	15.7 ± 2.8	22.2 ± 9.5	105 ± 94	
24	26.2 ± 9.9	29.8 ± 16.2	76.5 ± 23.5	436 ± 198	
	0.0003	0.0001	0.0001	0.0001	p^1^

For “Peak K_o_“ and “t_50_”: At 37°C, n = 7, with 45 contractions analyzed. At 24°C, n = 6, with 38 contractions analyzed. Data were measured under no flow conditions. For “Burst duration” and “Time between contractions”, data were obtained under flow conditions on the same tissue at both temperatures, first at 24° then at 37°C. n = 3, with 5 contractions analyzed for each experiment at both 24°C and 37°C.

1. For peak “K_o_“ and “t_50_” data were unpaired and p values were obtained using the 2-tailed t-test. For “Burst duration” and “Time between contractions”, p values were obtained using the paired t-test.

Rat myometrium also expresses spontaneous contractions at room temperature. Except for one experiment where we were able to raise the temperature and maintain the signal from the K-sensitive electrode, different tissues were studied at 24°C. We performed 6 experiments and analyzed 38 contractions at 24°C. At 24°C rises in K_o_ associated with bioelectrical activity were also observed, but peak K_o_ values were significantly greater than at 37°C (26.2±9.9 vs. 18.8±5.9 mM; p = 0.0003).

### Normalization of K_o_ at 37° and 24°C

As demonstrated in Figures 2 and 5, elevations in K_o_ persist well into the period between contractions. We use the term “normalization” to refer to the process where K_o_ returns to ∼5 mM before the start of the next contraction. Because rises in K_o_ were not observed after completion of the burst, we used the last spike to mark the beginning of the decay, even if the peak K_o_ did not occur at that time. By using this method of analysis, we were able to eliminate confounding effects due to varying burst durations and minimize the effects of the small irregular fluctuations of K_o_ that were often observed during the peak of the contraction.

**Figure 7:**
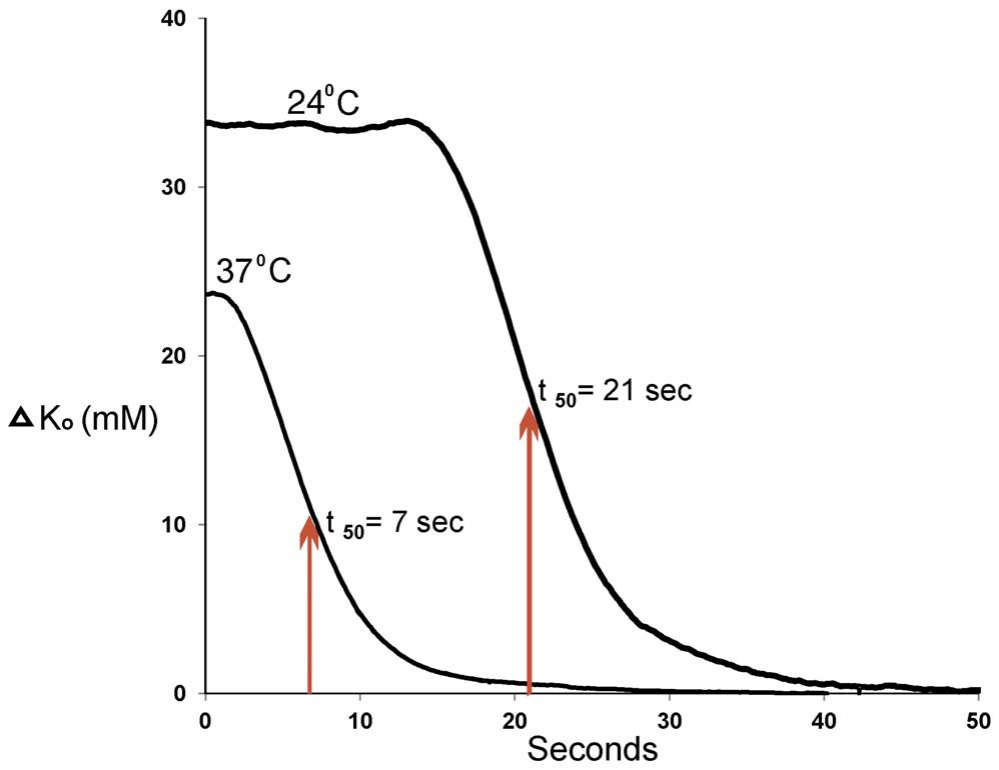
Normalization of ΔK_o_ at 37°C and 24°C. These data were recorded in a single experiment, initially at 24°C, then 37°C, without repositioning the K-sensitive electrode. Time is measured from the last spike of the burst. t_50_ values were obtained by measuring the time required for ΔK_o_ to decay to half the ΔK_o_ value measured at the last spike. Data obtained without bath flow.

To quantify the rate of normalization, we converted K_o_ values to changes in K_o_ (ΔK_o_ = K_o_ – 5), and then plotted ΔK_o_ vs. time (Fig. 7). In one experiment the data were obtained from a single tissue first at 24°C, then at 37°C, without moving the electrode. At 37°C, the decay of ΔK_o_ began soon after the end of the burst. At 24°C, a moderate delay was observed prior to the decay. Since the decay at 24°C did not appear to be a first-order mechanism, we approximated the normalization rate of K_o_ by the time it took for ΔK_o_ to fall to half the ΔK_o_ value observed at the last spike of the bioelectrical activity (t_50_). Because t_50_ was very sensitive to motion artifacts, we performed these experiments without using bath flow.

In 6 experiments at 24°C and 7 experiments at 37°C, using different tissues, t_50_ was more than twice as long at 24°C than at 37°C (unpaired t-test analysis, Table 1: 29.8±16.2 vs 15.7±2.8 seconds; p = 0.0001).

### Contraction frequency at physiological and reduced temperatures

The period is measured as the time between the first spike of the bioelectrical signal of one contraction and the first spike of the next. Additionally, the bioelectrical signals allow separation of the contraction period into two physiologically distinct periods - the duration of the contraction (burst duration), and the time between contractions (time between the last spike of one contraction and the first of the next).

Rat myometrium spontaneously contracts at 24°C, and raising the temperature shortens the period. However, we are unaware of published reports indicating if the period is shortened by the change of burst duration, change of time between contractions, or both. This series of experiments was performed to address that question, and determine the relationships of peak K_o_ and t_50_ to burst duration and the time between contractions.

It is difficult to directly compare contraction frequency and burst duration among tissues because different tissue strips express large variations of contraction patterns. To directly determine the effects of changing temperature, we first obtained stable contractions and recorded bioelectrical activity 24°C. We then increased the temperature to 37°C while continuing to record bioelectrical activity. Data were then compared using the paired t-test.

Three experiments were performed, and 5 contractions were analyzed for each tissue at each temperature. As expected, raising the temperature shortened the contraction period. More importantly, we found that both burst duration and the time between contractions were shorter at 37°C. Interestingly, raising the temperature shortened both parameters in approximately the same proportion (Table 1).

## Discussion

The primary finding of this work is that large rises in K_o_ occur during myometrial contractions, and K_o_ elevations persist well into the period between contractions. The second finding is that K_o_ begins to rise ∼0.6 seconds after the beginning of the action potential. This indicates two things: 1. Electrical activity causes K_o_ rises rather than vice versa; 2. Tissue excitability may be dynamically modulated by phasic rises in K_o_.

The values for peak K_o_ we observed are, to our knowledge, the largest reported in any muscle tissue under any conditions. Three factors associated with myometrium likely contribute to this. First, most of the calcium responsible for raising intracellular free calcium originates outside the cell, and then enters the cell via transmembrane calcium currents during the depolarization. This is in contrast to cardiac and skeletal muscle systems, which predominately cycle calcium into and out of intracellular stores. Second, intracellular calcium is highly buffered in myometrium[19]. Therefore, to raise intracellular free calcium, enough calcium has to be brought into the cell to overcome the cytosol's buffer capacity. Third, the myocytes of myometrium are closely packed with narrow restricted spaces between cells. In rodent, the myocytes are closely packed in sheets and in human they are closely packed in bundles. Recently Smith *et al* [20] confirmed by electron microscopy that the space between human myocytes is no greater than 1 µm.

Putting these factors together, we propose the following mechanism for myometrium: To express a contraction, large inward calcium currents must be generated to overcome the intracellular calcium buffering. In order to maintain the membrane potential at or below 0 mV, the inward calcium current must be balanced by an outward current. The outward currents are predominately K^+^ currents, which move moderate amounts of K^+^ from the inside of the cell into the restricted space between cells. A number of K^+^ channels could participate, such as Kv[21], inwardly rectifying K^+^ (Kir) channels[22], or a variety of Ca^2+^-activated K^+^ channels[23]. Because the volume of the restricted space is very small and K^+^ is not buffered, K_o_ rises.

To assess the feasibility of our proposed mechanism, a semi-quantitative assessment can be performed. For a first-order approximation, it is necessary to consider the outward currents, the duration of the currents, and the restricted volume. In isolated myocytes[24] measured at 0 mV, outward currents densities are on the order of 10 µA/cm^2^, and we will consider a 30 second contraction duration. We will not consider diffusion of K_o_ away from the plasma membrane at this time because myometrial myocytes are tightly packed, and we will assume that most myocytes are fully sheathed by the 1 µm restricted space.

Considering a square micron at the surface of a myocyte, a current density of 10 µA/cm^2^ lasting 30 seconds will carry a total charge of ∼3 picocoulombs/ µm^2^. Since the charge is carried into a 1 µm restricted space, the corresponding volume is 1 µm^3^. Converting coulombs to moles and µm^3^ to liters, yields 30 mM. This calculation is only a first-order approximation since it does not consider mechanisms that return K_o_ back into the myocytes (which would reduce peak K_o_), but it also does not consider that adjacent myocytes simultaneously move K^+^ into the same restricted space (which would increase peak K_o_). However, this assessment does demonstrate that significant rises of K_o_ are feasible.

Because of the novelty of our findings and this being the first application of K-sensitive microelectrodes in myometrium, we investigated the possibility that our results are merely artifacts cause by extraneous conditions, such as tissue motion. Four experimental techniques were used to test for artifacts, all of which indicated that the voltage changes we observed were minimally affected by artifacts. First, we fixed both ends of the tissue to minimize movement, then substituted non-K-sensitive electrodes for K-sensitive electrodes (Fig. 3). This resulted in small negative voltage deflections (<5 mV) that quickly recovered by the end of the contraction. Second, we fabricated double-barreled electrodes (one K-sensitive, one open) and simultaneously recorded two electrical channels with force. One electrode was used for sensing K_o_ and the other used in the DC mode to sense potential changes caused by tissue motion, transmembrane currents, or streaming potentials. The key finding was that voltages measured only a few microns from the tip of the K-sensitive electrode did not mimic rises in K_o_ (Fig. 5). Voltages were again observed to be negative-going, although they tended to be larger than found with the tissues fixed at both ends. The greater magnitude of these voltages (occasionally approaching−20 mV) may be due to the greater motion inherent to using a force transducer.

For a third control experiment, we mechanically stretched the tissue 1 mm and found that the artifacts recorded from the K-sensitive electrode were very large, but also negative (Fig. 6). Importantly, the artifacts produced in this manner rapidly decayed to baseline and did not demonstrate a long-lasting positive voltage signal. Experiments performed at reduced temperature provide the fourth control. Comparing data obtained at 37° and 24°C (Table 1), peak K_o_ was significantly increased and t_50_ was significantly lengthened, effects that would be difficult to explain if signals from the K-sensitive electrodes were electrical artifacts.

Lastly, there was the possibility that with each contraction, tissue movement across the electrode tip physically disrupts some cells, which release K^+^, and by that mechanism causes contraction-associated rises in K_o_. However, as noted above, after grossly moving the tissue (Fig. 6) the K-sensitive electrode did not display a rebound positive deflection that would suggest damage-induced elevations in K_o_. Finally, the repeatability of the magnitude of signals from contraction-to-contraction (Fig. 2), and the delay between local electrical activity and the start of the K_o_ signal (∼600 msec in the double-barreled experiment), strongly suggest the signals from the K-sensitive electrodes are not the result of tissue disruption.

Taken together, our investigations of motion artifacts indicate that tissue movement likely results in an underestimation of K_o_ rises. Our data also suggest that the magnitude of the electrical artifacts is partly related to the magnitude of tissue movement. There were two possible techniques to compensate for motion artifacts. First, was to minimize tissue motion by securing the tissue at both ends and performing the experiments without bath flow. As demonstrated above, we observed <5 mV artifact with this technique. Additionally, we could use double-barreled electrodes and subtract the DC signal of the non-K-sensitive electrode from the K-sensitive electrode. The second method introduces additional approximations because it is difficult to match the impedances of the two halves of the double-barreled electrodes, and subtracting signals cannot be made without several assumptions and scaling. Because using the double-barreled electrode yields only a small benefit (if any), quantitative measurements of K_o_ were undertaken by fixing the tissues at both ends and using single K-sensitive electrodes.

In this work we have defined the term “K_o_” to mean the concentration of K^+^ in the extracellular space. Within the extracellular space are the interstitial space, the vascular space, and the perivascular space[25]. For myometrium, there are additional spaces in the connective tissue between muscle layers (rat) or fasciculata[26] (human). Thus, it is feasible that the K-sensitive electrode tip could have been placed in any of these spaces.

The lack of pressurization of the vessels in these experiments, and the relatively small volume of the vascular space compared to cellular space, suggest that the K-sensitive electrodes were routinely placed into the smooth muscle component of the tissue. Since the only reasonable source of K^+^ is from the myocytes, electrode placement in the connective tissue between muscle layers on the rat, or in the spaces between fasciculate in human, would not be expected to produce large signals. Because K_o_ began to rise soon after the start of electrical activity, it is likely that a more specific source of K^+^ was from regions where neighboring myocytes expressed electrical activity – e.g. within layers or bundles. Therefore, it is likely that our observations reflect K_o_ in the interstitial space.

To reduce electrical artifact, some experiments were performed without bath flow. Under these conditions, the tissue could have experienced progressive acidosis or other adverse metabolic events with increasing time. However, under both flow and no-flow conditions, transient rises of K_o_, were observed over many minutes to hours. In experiments lasting more than one hour, K_o_ between contractions was the same as bath K^+^. This indicates that K_o_ did not build up in the tissue over time. Taken together, or data suggest that rises of K_o_ were not highly dependent on bath flow.

These dynamic changes in K_o_ in myometrium are the first to be directly observed without external pacing in any muscle tissue. To our knowledge, the values for peak K_o_ we report here are also the largest reported in any muscle tissue. The magnitude of these values suggests that K_o_ participates in modulating myometrial contractility during normal function.

While we used the temperature dependence data to argue against the significance of artifacts, the primary purpose behind performing experiments at different temperatures was to investigate the associations among K_o_, contraction duration, and contraction frequency. Responses of K-sensitive electrodes were unchanged by changing between 24° and 37°C (see Figure S1). Compared with 37°C, larger values of peak K_o_ and slower normalization of K_o_ between contractions were found at 24°C (Table 1). The greater than 2-fold slowing of rate of K_o_ normalization at reduced temperature suggests the mechanism predominately involves a metabolic process, likely Na^+^, K^+^ exchange[27]. Temperature-dependent inhibition of Na^+^, K^+^ exchange may also contribute to peak K_o_ at 24°C being larger than at 37°C.

Raising K_o_ changes the K^+^ Nernst potential, depolarizes the tissue and causes smooth muscle to contract. Through this mechanism, the large rises in K_o_ we observe during the force-producing phase of the action potential may contribute to lengthening the duration of each contraction.

On the other hand, modest elevations of bath K^+^ (6 to 16 mM)[28] causes relaxation of vascular smooth muscle. Values of K_o_ in this range could activate inward rectifying (Kir) potassium channels[10], which would provide hyperpolarizing currents. In myometrium, elevated K_o_ enhances activity of the electrogenic Na^+^, K^+^ exchanger[11], which also favors hyperpolarization. Therefore, the modest K_o_ elevations that persisted between myometrial contractions would tend to hyperpolarize the tissue and prolong the time between contractions.

We should emphasize that our data were obtained without external stimulation, and that the K_o_ rises we report here were attributable to phasic contractions that reasonably mimic normal physiological conditions. K_o_ rises likely modulate the function of all the cells that share the same extracellular space. In this sense, K_o_ can be seen as contributing to the syncytial behavior of the tissue through a gap junction-independent mechanism. Cells that reside within the interstitial space, such as Cajal-like cells[29], may also be subject to the influence of changing K_o_. We propose that K_o_ metabolism provides a novel mechanism for moment-to-moment modulation of phasic myometrial contractions.

## Supporting Information

Figure S1 **Responses of K-sensitive electrodes at 24° and 37°C.** At 24°C, three K-sensitive electrodes were zeroed in 2.5 mM KCl solution, and then tested at 5, 10, 20 and 40 mM. Each electrode was immediately transferred to 2.5 mM KCl solution, re-zeroed, and retested at each KCl concentration at 37°C.
(TIF)

## References

[1] Anderson NC Jr (1969) Voltage-clamp studies on uterine smooth muscle. J Gen Physiol 54: 145–165. 5796366 10.1085/jgp.54.2.145 PMC2225926

[2] Sanborn BM (2000) Relationship of ion channel activity to control of myometrial calcium. J Soc Gynecol Investig 7: 4–11. 10.1016/s1071-5576(99)00051-9 10732311

[3] Somlyo AP, Somlyo AV (1994) Signal transduction and regulation in smooth muscle. Nature 372: 231–236. 7969467 10.1038/372231a0

[4] Bursztyn L, Eytan O, Jaffa AJ, Elad D (2007) Modeling myometrial smooth muscle contraction. Ann N Y Acad Sci 1101: 110–138. 17303825 10.1196/annals.1389.025

[5] Aslanidi O, Atia J, Benson AP, van den Berg HA, Blanks AM, et al (2011) Towards a computational reconstruction of the electrodynamics of premature and full term human labour. Prog Biophys Mol Biol 107: 183–192. 21777604 10.1016/j.pbiomolbio.2011.07.004

[6] Taggart MJ, Wray S (1998) Hypoxia and smooth muscle function: key regulatory events during metabolic stress. J Physiol 509 (Pt 2): 315–325. 10.1111/j.1469-7793.1998.315bn.x PMC2230985 9575282

[7] Singer W, Lux HD (1973) Presynaptic depolarization and extracellular potassium in the cat lateral geniculate nucleus. Brain Res 64: 17–33. 4360876 10.1016/0006-8993(73)90168-6

[8] Somjen GG (2002) Ion regulation in the brain: implications for pathophysiology. Neuroscientist 8: 254–267. 12061505 10.1177/1073858402008003011

[9] Davis MJ (2012) Perspective: physiological role(s) of the vascular myogenic response. Microcirculation 19: 99–114. 21895845 10.1111/j.1549-8719.2011.00131.x

[10] Edwards G, Dora KA, Gardener MJ, Garland CJ, Weston AH (1998) K+ is an endothelium-derived hyperpolarizing factor in rat arteries. Nature 396: 269–272. 9834033 10.1038/24388

[11] Burns WR, Cohen KD, Jackson WF (2004) K+-induced dilation of hamster cremasteric arterioles involves both the Na+/K+-ATPase and inward-rectifier K+ channels. Microcirculation 11: 279–293. 15280082 10.1080/10739680490425985 PMC1382024

[12] Coats P, Johnston F, MacDonald J, McMurray JJ, Hillier C (2001) Endothelium-derived hyperpolarizing factor: identification and mechanisms of action in human subcutaneous resistance arteries. Circulation 103: 1702–1708. 11274000 10.1161/01.cir.103.12.1702

[13] Juel C, Pilegaard H, Nielsen JJ, Bangsbo J (2000) Interstitial K(+) in human skeletal muscle during and after dynamic graded exercise determined by microdialysis. Am J Physiol Regul Integr Comp Physiol 278: R400–406. 10666141 10.1152/ajpregu.2000.278.2.R400

[14] Kline RP, Morad M (1978) Potassium efflux in heart muscle during activity: extracellular accumulation and its implications. J Physiol 280: 537–558. 308540 10.1113/jphysiol.1978.sp012400 PMC1282675

[15] Kline RP, Kupersmith J (1982) Effects of extracellular potassium accumulation and sodium pump activation on automatic canine Purkinje fibres. J Physiol 324: 507–533. 7097608 10.1113/jphysiol.1982.sp014127 PMC1250720

[16] Obrocea GV, Morris ME (1998) Changes in [K+]o evoked by baclofen in guinea pig hippocampus. Can J Physiol Pharmacol 76: 148–154. 9635153

[17] Young RC, Bemis A (2009) Calcium-activated chloride currents prolongs the duration of contractions in pregnant rat myometrial tissue. Reprod Sci 16: 734–739. 19380901 10.1177/1933719109334965

[18] Young RC, Goloman G (2011) Mechanotransduction in rat myometrium: coordination of contractions of electrically and chemically isolated tissues. Reprod Sci 18: 64–69. 20713968 10.1177/1933719110379637

[19] Daub B, Ganitkevich V (2000) An estimate of rapid cytoplasmic calcium buffering in a single smooth muscle cell. Cell Calcium 27: 3–13. 10726206 10.1054/ceca.1999.0084

[20] Smith RD, Babiychuk EB, Noble K, Draeger A, Wray S (2005) Increased cholesterol decreases uterine activity: functional effects of cholesterol alteration in pregnant rat myometrium. Am J Physiol Cell Physiol 288: C982–988. 15613497 10.1152/ajpcell.00120.2004

[21] Smith RC, McClure MC, Smith MA, Abel PW, Bradley ME (2007) The role of voltage-gated potassium channels in the regulation of mouse uterine contractility. Reprod Biol Endocrinol 5: 41. 17980032 10.1186/1477-7827-5-41 PMC2186335

[22] Zaritsky JJ, Eckman DM, Wellman GC, Nelson MT, Schwarz TL (2000) Targeted disruption of Kir2.1 and Kir2.2 genes reveals the essential role of the inwardly rectifying K(+) current in K(+)-mediated vasodilation. Circ Res 87: 160–166. 10904001 10.1161/01.res.87.2.160

[23] Noble K, Floyd R, Shmygol A, Mobasheri A, Wray S (2010) Distribution, expression and functional effects of small conductance Ca-activated potassium (SK) channels in rat myometrium. Cell Calcium 47: 47–54. 19969350 10.1016/j.ceca.2009.11.004

[24] Shmygol A, Noble K, Wray S (2007) Depletion of membrane cholesterol eliminates the Ca2+-activated component of outward potassium current and decreases membrane capacitance in rat uterine myocytes. J Physiol 581: 445–456. 17331986 10.1113/jphysiol.2007.129452 PMC2075177

[25] Law RO (1982) Techniques and applications of extracellular space determination in mammalian tissues. Experientia 38: 411–421. 7044812 10.1007/BF01952615

[26] Young RC, Hession RO (1999) Three-dimensional structure of the smooth muscle in the term-pregnant human uterus. Obstet Gynecol 93: 94–99. 9916964 10.1016/s0029-7844(98)00345-7

[27] Clausen T (2003) Na+-K+ pump regulation and skeletal muscle contractility. Physiol Rev 83: 1269–1324. 14506306 10.1152/physrev.00011.2003

[28] Knot HJ, Zimmermann PA, Nelson MT (1996) Extracellular K(+)-induced hyperpolarizations and dilatations of rat coronary and cerebral arteries involve inward rectifier K(+) channels. J Physiol 492 (Pt 2): 419–430. 10.1113/jphysiol.1996.sp021318 PMC1158837 9019539

[29] Duquette RA, Shmygol A, Vaillant C, Mobasheri A, Pope M, et al (2005) Vimentin-positive, c-kit-negative interstitial cells in human and rat uterus: a role in pacemaking? Biol Reprod 72: 276–283. 15385413 10.1095/biolreprod.104.033506

