# Supplementary figures and images for: Phasic Oscillations of Extracellular Potassium (Ko) in Pregnant Rat Myometrium
Source: PLoS One. 2013 May 28;8(5):e65110. doi: 10.1371/journal.pone.0065110 (PMC3665820; doi:10.1371/journal.pone.0065110)

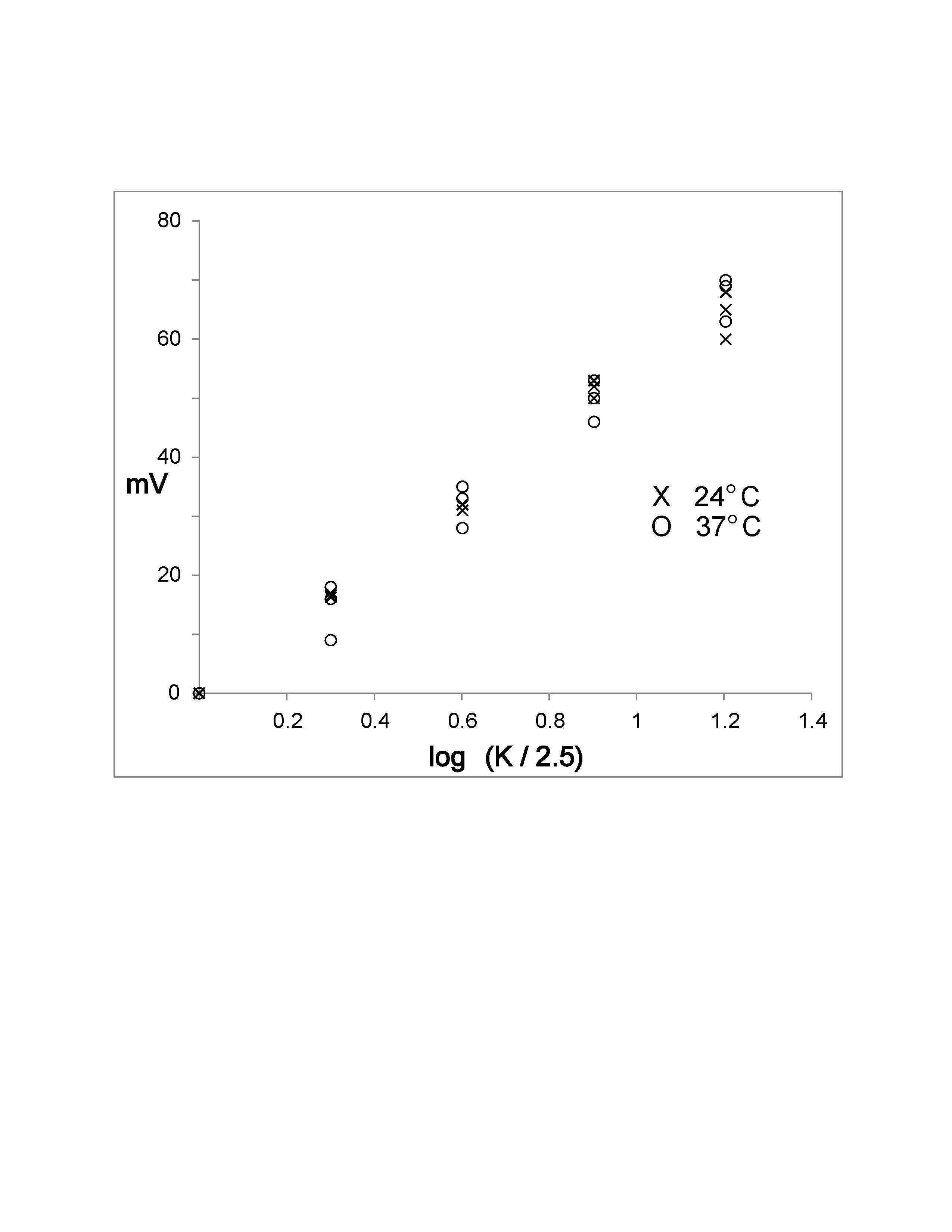

Supplement: Figure S1 — Responses of K-sensitive electrodes at 24° and 37°C. At 24°C, three K-sensitive electrodes were zeroed in 2.5 mM KCl solution, and then tested at 5, 10, 20 and 40 mM. Each electrode was immediately transferred to 2.5 mM KCl solution, re-zeroed, and retested at each KCl concentration at 37°C. (TIF) [file pone.0065110.s001.tif]
